# Extreme-Ultraviolet Excited Scintillation of Methylammonium Lead Bromide Perovskites

**DOI:** 10.1021/acs.jpcc.2c02400

**Published:** 2022-07-21

**Authors:** Maarten L.S. van der Geest, Lucie McGovern, Stefan van Vliet, Hanya Y. Zwaan, Gianluca Grimaldi, Jeroen de Boer, Roland Bliem, Bruno Ehrler, Peter M. Kraus

**Affiliations:** † Advanced Research Center for Nanolithography, Science Park 106, 1098 XG Amsterdam, The Netherlands; ‡ Center for Nanophotonics, AMOLF, Science Park 102, 1098 XG Amsterdam, The Netherlands; § Cavendish Laboratory, University of Cambridge,CB2 1TN Cambridge, United Kingdom; ∥ Institute of Physics, University of Amsterdam, Science Park 904, 1098 XH Amsterdam, The Netherlands; ⊥ Department of Physics and Astronomy, and LaserLaB, Vrije Universiteit, De Boelelaan 1105, 1081 HV Amsterdam, The Netherlands

## Abstract

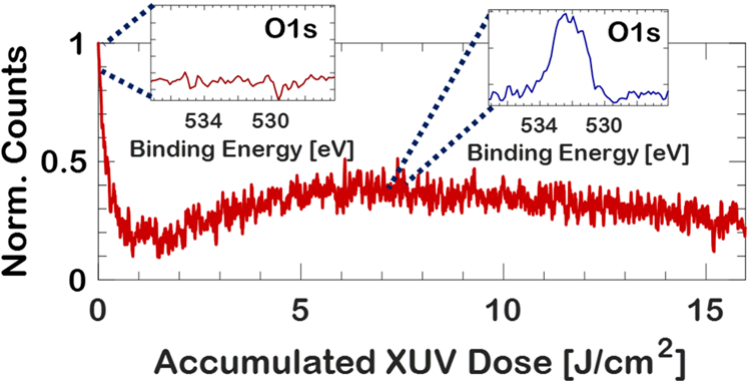

Inorganic–Organic lead halide materials have been recognized as potential high-energy X-ray detectors because of their high quantum efficiencies and radiation hardness. Surprisingly little is known about whether the same is true for extreme-ultraviolet (XUV) radiation, despite applications in nuclear fusion research and astrophysics. We used a table-top high-harmonic generation setup in the XUV range between 20 and 45 eV to photoexcite methylammonium lead bromide (MAPbBr_3_) and measure its scintillation properties. The strong absorbance combined with multiple carriers being excited per photon yield a very high carrier density at the surface, triggering photobleaching reactions that rapidly reduce the emission intensity. Concurrent to and in spite of this photobleaching, a recovery of the emission intensity as a function of dose was observed. X-ray photoelectron spectroscopy and X-ray diffraction measurements of XUV-exposed and unexposed areas show that this recovery is caused by XUV-induced oxidation of MAPbBr_3_, which removes trap states that normally quench emission, thus counteracting the rapid photobleaching caused by the extremely high carrier densities. Furthermore, it was found that preoxidizing the sample with ozone was able to prolong and improve this intensity recovery, highlighting the impact of surface passivation on the scintillation properties of perovskite materials in the XUV range.

## Introduction

Inorganic–Organic lead halide perovskites (APbX_3_, with A = methylammonium (MA, CH_3_NH_3_^+^), formamidinium (FA, CH(NH_2_)_2_^+^), X = Cl, Br, I) have come to the scientific forefront through their potential in many diverse applications, such as solar cell materials,^1^ memristors,^2,3^ and high-energy radiation detectors.^4^ This application potential arises from their favorable and unique properties, including high photoluminescence (PL) quantum efficiency,^5^ long charge carrier and exciton diffusion lengths,^6−8^ ion migration and associated memory effects,^9,10^ strong absorbance in the near IR/visible to ultraviolet range^11^ and the hard X-ray range^12^ as well as facile and cheap solution-based fabrication into both single-crystal and polycrystalline structures.^13^ It is therefore not surprising that lead halide-based perovskites, both fully inorganic and organic–inorganic, were reported as promising materials for X-rays (>1 keV) and high-energy particle detection.^14,15^ Of particular interest for high-energy photon and particle detectors are the excellent downconversion quantum efficiencies, radiation hardness, and intrinsic tunable visible wavelength emission through mixing halides and adding interstitial atoms in appropriate ratios.^4,15^ Equally important, however, compared to traditional radiation detection materials such as CsI:Tl, lead halide perovskites have a very low production cost.^4^ Combined, these properties could make for excellent indirect scintillators. In an indirect scintillator, high-energy radiation or particles excite photoelectrons, which in turn excite charge carriers that eventually decay to emit detectable photons. A good indirect XUV scintillator has a high conversion efficiency of excitation photons to carriers and of carriers to emitted photons and can maintain this conversion efficiency as the dose of ionizing XUV radiation accumulates. The number of emitted photons versus accumulated dose is known as the photostability.^16^ The photostability of an indirect XUV scintillator is important because photobleaching causes the emission to decrease over time, making the scintillator less effective. Chemical and physical changes can cause either a decrease or an increase in photostability, as both can affect the number of emitting centers available.^4^

Lead halide perovskites were found to be effective intrinsic indirect scintillators for β-particles^17^ and γ-rays produced in positron emission tomography^18^ as well as for soft and hard X-rays.^12,15^ Little has been done to investigate whether this effectiveness extends to lower energy ionizing radiation. While performing well under hard X-rays,^14^ the performance of methylammonium lead bromide (MAPbBr_3_) under extreme ultraviolet (XUV) radiation (10–120 eV) exposure is therefore not extensively studied and is thus poorly understood. Understanding this behavior is critical for evaluating the performance of these perovskites as XUV scintillator, but it can also be critical, for instance, in space-based solar cells,^19^ where XUV radiation and low-energy particles originating from the sun form a potential damage source for photovoltaic materials, both conventional and those based on APbX_3_ perovskites.

Here, we report the investigation of the visible light emission of polycrystalline MAPbBr_3_ thin films excited by broadband XUV pulses. This allows us to assess the potential of MAPbBr_3_ thin films as indirect XUV scintillators. Radiation in the XUV spectral range is strongly absorbed by MAPbBr_3_ due to the ionizing energy of the radiation,^20^ which can induce rapid chemical and physical changes of the material. A major chemical change observed in the material surface was an oxidation reaction, resulting in passivation of the surface. The result of this oxidation is a spectral intensity recovery that counteracts the photobleaching, which otherwise leads to nonradiative recombination. The spectral intensity recovery is explored in detail, and we propose methods of using the oxidation to further improve the scintillation properties of MAPbBr_3_.

## Methods

### Thin-Film Preparation

The samples consist of MAPbBr_3_ spin-coated on quartz to a thickness of 180 ± 20 nm and a grain size of 200–500 nm, as estimated from the morphology in scanning electron microscopy micrographs in an earlier publication using the same recipe.^10^

Quartz substrates (12 × 12 mm^2^, with 1 mm thickness) were cleaned by ultrasonication for 15 min, followed by a cleaning and ultrasonication with detergent, deionized water, acetone, and isopropyl alchohol, and finally the cleaned substrates were further cleaned with an O_2_ plasma cleaner for 15 min. The MAPbBr_3_ perovskite precursor solution was prepared by dissolving 1.1 M of methylammonium bromide (98.0%, Sigma-Aldrich) and lead(II) bromide (PbBr_2_ > 98.0Z > 98.0%, TCI) with a 1:1 molar ratio into a 4:1 dimethylformamide (anhydrous, 99.8%, Sigma-Aldrich)/dimethyl sulfoxide (anhydrous, ≥99.9%, Sigma-Aldrich) solvent mix. The dissolution took place overnight on a hot plate at 60 °C. After having cooled, 100 μL of the MAPbBr_3_ precursor solution was spun onto the quartz substrates at 6000 rpm for 30 s in a nitrogen-filled glovebox. Fifteen seconds after the beginning of the rotation, 100 μL of chlorobenzene antisolvent (anhydrous, Sigma-Aldrich) was pipetted onto the substrate. After the spin-coating procedure, the substrates were annealed at 100 °C for 1 h. During all steps mentioned, the atmosphere inside the glovebox was maintained at an O_2_ level below 1 ppm. For the MAPbBr_3_ samples that were treated with ozone as discussed in the corresponding section, samples were exposed using the UV ozone cleaner (Ossila) at a temperature of 20 °C.

### Thin-Film Characterization

The X-ray photoelectron spectroscopy (XPS) analyses were performed with an HiPP-3 spectrometer (Scienta Omicron) using a monochromatic aluminum Kα source (1486.6 eV, 20.0 mA, 14 kV). The HiPP-3 analyzer is used with a 0.8 mm cone and a slit setting of 1.0 mm. An XPS data analysis was done through the fitting of Voigt profiles and performed using KolXPD. Surface charging corrections were applied based on the apparent binding energy of the Br 3d_5/2_ peak.^21^ Relative peak areas were obtained by dividing the peak areas of peaks obtained with the same pass energy by their tabulated 1486.6 eV photoemission cross sections and setting the area of the Pb 4f peak to one.

Ultraviolet–Visible (UV–vis) spectroscopy measurements were conducted with a UV-2600 UV–vis spectrophotometer (Shimadzu) with a 1 nm step size and measured against a blank quartz substrate.

X-ray diffraction (XRD) measurements were conducted using a D2 Phaser (Bruker) with a copper Kα_2_ (8027.8 eV) source, with a low-pass angle pinhole to ensure that only the area of interest is measured and not the entire spot size of the XRD beam. Because of this, the spectra have to be integrated over many hours to achieve the required signal-to-noise ratio.

### Extreme-Ultraviolet Excited Luminescence with a High-Harmonic Source

**Figure 1:**
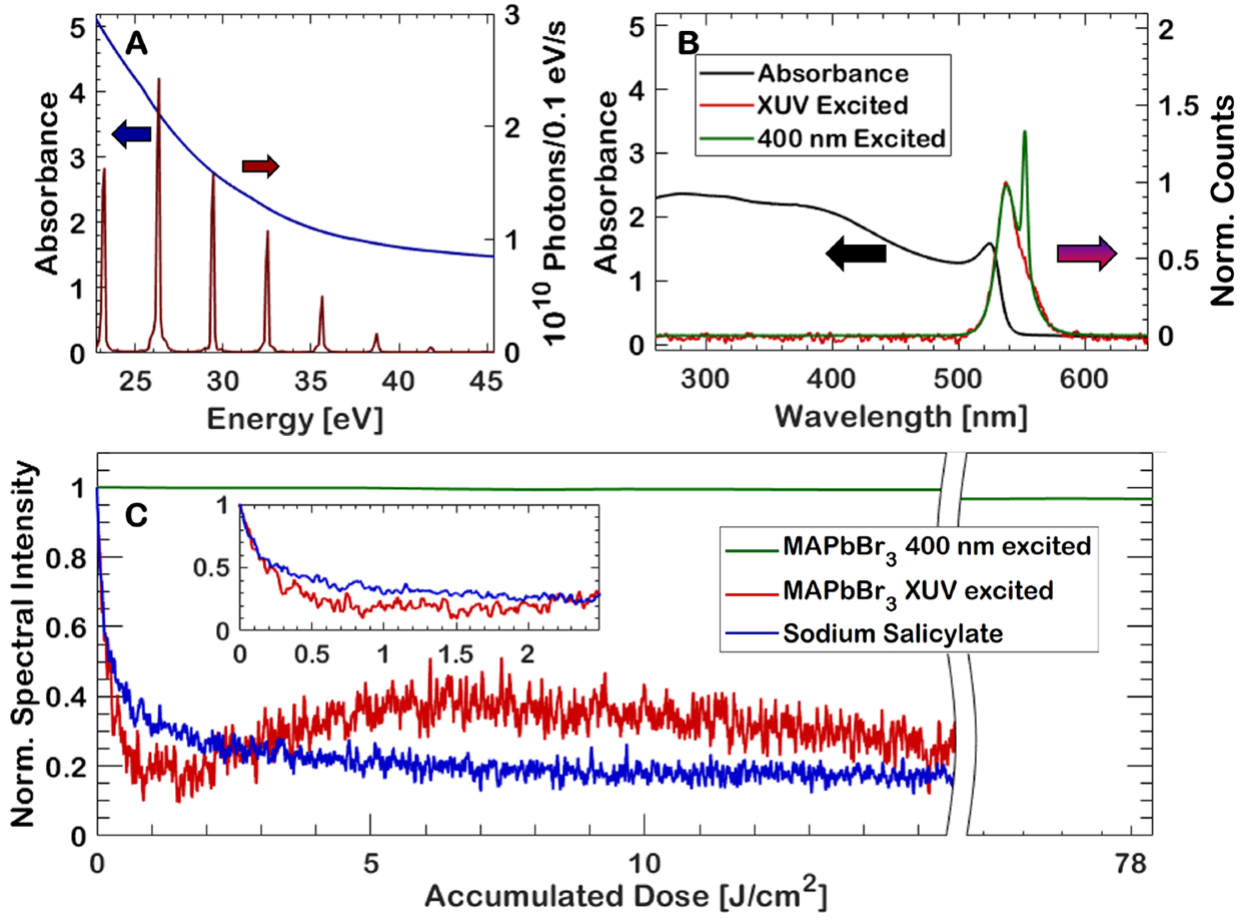
(A) Calculated absorption spectrum (blue) of 180 nm MAPbBr_3_ based on atomic scattering factors and the reported density, and XUV spectrum (red) from HHG, with a calibrated intensity axis in number of photons per second per 0.1 eV. (B) UV–Vis absorption (black) and emission spectrum when excited by broadband XUV radiation (red) and 400 nm pulsed radiation (blue). The feature at 560 nm originates from random lasing in the polycrystalline MAPbBr_3_ thin film. (C) Normalized spectral emission intensity as a function of accumulated dose on target in the case of MAPbBr_3_ excited with 400 nm (green), XUV (red), and sodium salicylate (blue), also excited by XUV pulses.

The perovskite thin film is exposed to XUV radiation using a table-top setup. A more detailed overview of this setup and its characteristics is given in a previous publication.^22^ Briefly, 1.1 mJ is split from the output of a 2 kHz titanium:sapphire (Ti:Sa) laser (Solstice ACE, Spectra Physics, 800 nm, 35 fs, 7 W) and focused into a noble gas target in vacuum. This generates broadband XUV radiation through the high-order harmonic generation (HHG) process.^23−26^ A representative spectrum generated at peak intensities of ∼1.5 × 10^14^ W/cm^2^ is shown in Figure 1A. The XUV radiation is refocused with a toroidal mirror onto the sample mounted on a sample stage. The pulse energy is estimated to be ∼0.5 nJ/pulse on target.^22,27^ The spot size of the XUV on the sample is ∼45 μm full-width at half-maximum (fwhm), measured with a knife edge. Together this yields a single pulse fluence of 26 μJ/cm^2^. An accumulated broadband XUV dose of 15 J/cm^2^ is thus achieved within 5 min using our table-top source. Different excitation wavelengths between 220 and 2400 nm are generated in a wavelength-tunable optical parametric amplifier (OPA) pumped by 1.3 mJ from the same Ti:Sa laser. This was used to compare the XUV-excited emission with emission excited by longer-wavelength pulses, in this series of experiments between 260 and 400 nm center wavelength. The spot size of the output of the wavelength-tunable OPA is ∼150 μm fwhm at the sample position, measured with a knife edge.

The emission from the sample is collimated by a short focal length parabolic mirror and guided out of the vacuum chamber into a streak camera (C10910, Hamamatsu) for time-resolving the decay kinetics or into a spectrometer (Maya 2000 PRO, Ocean Optics) to follow the emission and bleaching on longer time scales (up to tens of minutes). The sample is raster-scanned to expose larger areas where required.

## Results and Discussion

### XUV Photostability

MAPbBr_3_ shows intense luminescence when excited with XUV radiation, as expected due to earlier reported hard X-ray excited luminescence in MAPbBr_3_.^14^ The XUV-excited visible luminescence spectrum is shown as the red curve in Figure 1B and peaks at ∼540 nm. Because of XUV-induced photobleaching the intensity of this luminescence decreases rapidly over time (Figure 1C). Using atomic scattering factors,^20^ we find that the absorption of XUV radiation in the range between 20 and 45 eV is particularly strong for MAPbBr_3_ due to the proximity of the photon energies to the ionization threshold and due to its constituent heavily absorbing elements and having a high density of ∼3.5 g/cm^3^.^28^ The XUV absorbance of a 180 nm thin film of MAPbBr_3_ is depicted in blue in Figure 1A. When matched with a typical XUV spectrum from our HHG-based XUV source (Figure 1A, in red), one can see that the absorbance is highest where the XUV photon flux is likewise highest. The XUV absorbance is larger than anywhere within the UV–vis absorption range, which is shown in the black curve in Figure 1B, and also larger than anywhere else in the X-ray range.^20^ A calculation with Beer–Lambert’s law, using the known XUV spectrum, suggests that 99% of all photons are absorbed within the first 75 nm of the MAPbBr_3_ thin film, as elaborated on in the Supporting Information. In addition, a single XUV photon has more than 10 eV excess energy, when compared to visible or (D)UV photons, with which it can indirectly excite multiple carriers through secondary electron cascades, further contributing to a high carrier density.^29^ This yields an estimated carrier density of between 6.2 × 10^19^ (at one carrier per XUV photon) and 6.7 × 10^20^ cm^–3^ (maximum discrete amount of carriers per XUV photon). In the surface region even higher carrier densities are estimated (between 2.3 × 10^20^ and 2.4 × 10^21^ cm^–3^). See the Supporting Information for details on the calculation. The large estimation range originates from the unknown XUV photon to carrier conversion factor. Figure 1C depicts the integrated and normalized spectral emission intensity of a single-pulse excitation fluence of 8 mJ/cm^2^ excited by 400 nm pulses (green curve), which shows a very small decrease of the emission intensity for large exposure doses (2% decrease for 78 J/cm^2^ accumulated dose), despite an estimated carrier density of (6.7 ± 0.2) × 10^20^ cm^–3^. This stands in sharp contrast to the more than 75% decrease in emission intensity for XUV excitation with an accumulated dose of 15 J/cm^2^ and 26 μJ/cm^2^ single-pulse fluence. This compares favorably with several plastic scintillators as can be seen in the Supporting Information, Figure S2.

As the estimated surface carrier densities are very high in both cases, but nevertheless comparable, the rapid photobleaching has to be explained in a different manner. The major difference between the two is that, in the case of XUV excitation, the photons have a lot of excess energy with which to trigger chemical reactions at the surface and in this way bleach the emitting state. This high carrier density accelerates reactions responsible for photobleaching and modifies the surface, where most of the XUV-excited emission originates, thus quenching the luminescence. The resulting carrier density distribution does not affect the shape of the emission spectrum, as can be seen in Figure 1B, although the sharp amplified spontaneous emission (ASE) feature that results in random lasing at 560 nm is not clearly visible when MAPbBr_3_ is excited with XUV radiation, despite our carrier density estimates suggesting that it should be visible.^30^ The reason for the absence of the ASE is unknown. It is likely a combination of inefficient pumping of gain channels due to the large separation between excitation and emission wavelength, making it less likely to form the required conditions for ASE, in combination with the previously mentioned rapid photobleaching making the conditions even more unfavorable.

The photobleaching of the emission of MAPbBr_3_ as a function of accumulated dose shows anomalous behavior, not seen in the commonly used XUV scintillator sodium salicylate (Figure 1C, blue curve).^31^ In the case of sodium salicylate, the emission intensity drops rapidly after receiving an accumulated dose of just 2 J/cm^2^, due to high excited-state densities, triggering photobleaching of the emitting state.^32^ This is followed by a slow further photobleaching as the remaining emitting states within the excited sample volume are also bleached. Similar to sodium salicylate, the XUV-excited PL intensity initially rapidly decreases in MAPbBr_3_, as seen in the red curve in Figure 1C. This is however followed by a remarkable recovery of the PL intensity. After receiving three times the dose it received at its lowest PL intensity (reaching 15% of its initial value), the emission recovers to 40% of the initial intensity. This remarkable recovery of PL intensity does not correspond to a change in line shape of the emission spectrum. The initial emission spectrum only shows an increase or decrease as a function of accumulated dose. Intermittent radiation exposure, where the XUV source was turned on and off by blocking the generating fundamental beam with a shutter, did not affect the observed emission intensity recovery in terms of minimum or maximum (Figure S1 in the Supporting Information), as the maximum was achieved at approximately the same accumulated dose. We hypothesize that the reason for this increase of emission intensity over time, after the initial rapid photobleaching, is a comparatively slow oxidation reaction that takes place at the surface, stimulated by the continuous XUV exposure, resulting in surface passivation. This hypothesis is verified in another section, vide infra, where we characterize an area of a pristine sample that has been exposed to ∼10 J/cm^2^ of accumulated XUV radiation. Passivation through ozone oxidation was reported to increase emission intensity in the case of 50 keV hard X-ray excitation of a single crystal,^14^ where it was attributed to ozone treatment leading to a reduced trap state density at the surface. Trap states are a major contributor to the reduced external quantum efficiency due to their ability to prevent radiative recombination of the excited carriers.^29,33^ In the following sections we confirm this oxidation-induced photostability by time-resolved luminescence and postexposure characterization.

### Time-Resolved Luminescence under XUV Exposure

**Figure 2:**
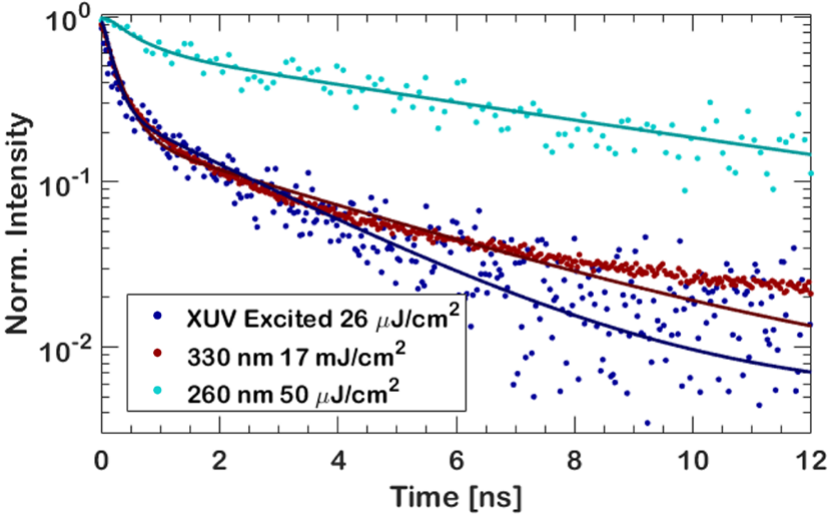
Comparison of the PL decay of MAPbBr_3_ when excited with broadband XUV radiation (blue) with a single-pulse fluence of 26 μJ/cm^2^, 330 nm excitation with 17 mJ/cm^2^ (red), and 260 nm excitation with 50 μJ/cm^2^, including fits.

The decay kinetics of the emission are strikingly affected by the excitation wavelength, as shown in Figure 2. The decay kinetics of an XUV-excited MAPbBr_3_ thin film with a single-pulse fluence of only 26 μJ/cm^2^ (blue) is comparable to the decay kinetics of a sample excited at a wavelength of 330 nm with a fluence of 17 mJ/cm^2^ (red), despite having a factor of 650 times lower fluence. This is also in stark contrast to the much slower decrease observed when exciting the thin film with a more comparable fluence of 50 μJ/cm^2^ at a deep ultraviolet (DUV) wavelength of 260 nm (cyan). Fitting a biexponential decay to the time-resolved, 260 nm excited emission yields τ_1_ = 0.56 ± 0.2 ns and τ_2_ = 7.7 ± 0.8 ns with nearly equal proportionality constants of α_1_ = 0.49 and α_2_ = 0.51. The reason for this contrasting behavior is again the high carrier concentrations, as elaborated on in the previous section, that are excited at the surface, which in turn results in rapid quenching of the emission. At such high carrier concentrations, trimolecular recombination pathways become relevant, but the radiative emission should still be approximately biexponential.^34^ One can thus fit a biexponential to the start of the XUV-excited PL decay and get a reasonable fit. This yields decay constants τ_1_ = 0.18 ± 0.02 ns and τ_2_ = 2.6 ± 0.2 ns with proportionality constants α_1_ = 0.83 and α_2_ = 0.17, in rough agreement with the decay times of cesium lead bromide composites excited with γ-rays.^18^ The high-fluence, 330 nm-excited decay similarly has a rapid decay with decay constants τ_1_ = 0.27 ± 0.02 ns and τ_2_ = 3.8 ± 0.2 ns with proportionality constants α_1_ = 0.88 and α_2_ = 0.12, but, as mentioned, an 800 times higher fluence is required to achieve this accelerated decay for 330 nm excitation. The carrier density in the case of the high-fluence 330 nm excitation is 9.8 × 10^20^ cm^–3^, which is in the same order of magnitude as the high estimate for XUV excitation. See the Supporting Information for details on the carrier density calculation. As is apparent from the fits in Figure 2, there is a long-term effect that is not properly captured by a simple biexponential fit at very high intensities, resulting in underfitting in the case of both the 330 nm and XUV-excited emissions. A more in-depth treatment of the PL from MAPbBr_3_ thin films when excited in the XUV range or at high fluences is necessary to fully appreciate the reason for a simple biexponential model being insufficient, which is beyond the scope of this investigation.

### Passivation through XUV Exposure

**Figure 3:**
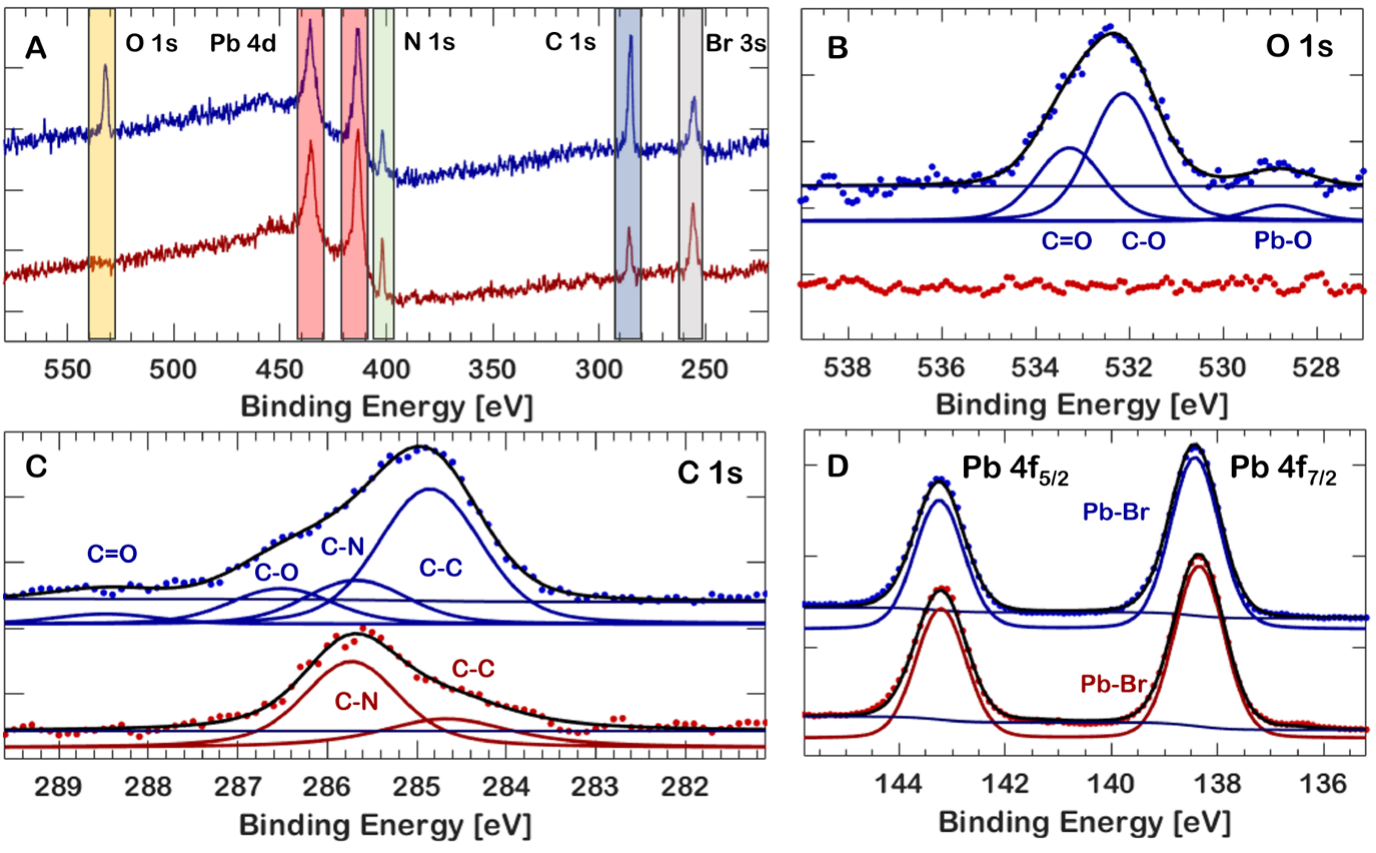
XPS spectra of exposed (blue) and pristine (red) surfaces of an MAPbBr_3_ thin film. (A) Survey spectrum with labeled peaks, showing the appearance of an O 1s peak and a strong shape change in the C 1s peak. (B) The O 1s peak of a pristine and exposed part of the sample. (C) The C 1s peak of exposed and pristine MAPbBr_3_ shows the formation of oxidized carbon species, in addition to a strong increase in the amount of C–C bonded carbon at low binding energies. (D) The Pb 4f peak, showing no change.

As the photostability experiments indicate, XUV exposure causes a chemical reaction to occur at the surface, which causes the beneficial emission recovery (Figure 1C). Specifically, we observe oxidation of the polycrystalline MAPbBr_3_ films due to the water layer that is present on virtually all surfaces, even in high-vacuum systems.^35^ It is reported that MAPbBr_3_ reacts with water in ambient air over time,^36^ so a reaction with water, especially when activation energy is provided through high-energy photons,^21^ is not unexpected. We exposed 1 mm^2^ of an MAPbBr_3_ thin-film sample to a dose of ∼10 J/cm^2^. XPS measurements of the unexposed and exposed part verify that oxidation of the surface has taken place. This is shown in Figure 3A, where the XUV-exposed area of a sample (blue curve) shows an O 1s feature, whereas this peak is absent in the unexposed part of the sample (red curve). The full XPS spectrum of the unexposed and exposed area of the MAPbBr_3_ can be seen in Figure S3A in the Supporting Information. Ratios between spectrally integrated XPS peaks are summarized in Table 1. As lead does not outgas or otherwise leave the sample,^21,36^ ratios are normalized to the Pb 4f peak area. These ratios are indicative of the elemental composition of the surface and deviate from the expected ratio based on the bulk formula. For the pristine samples, the ratios are comparable to previously reported elemental compositions.^21,36^ A closer look at the O 1s peak in Figure 3B shows that at least three species have formed, one being significantly lower in binding energy than the others. We shall look more closely at the two large ones first. Both species are oxidized forms of carbon, as lead oxide would be expected at lower binding energies. This is further confirmed by there being no noticeable shift or broadening in the Pb 4f peak (Figure 3D) and the amount of bromine relative to lead not changing, as can be seen in Table 1. Furthermore, the Br 3d peak does not show any noticeable change either (Figure S3D,E in the Supporting Information), which is not unexpected in an ultrahigh vacuum.^10,21^ The C–N peak in Figure 3C shows an apparent broadening after XUV exposure, caused by additional oxidized carbon species forming on the surface. These species are singly bonded (likely alcohols) and doubly bonded (ketones, aldehydes) carbon to oxygen, based on their binding energies and relative peak intensities in both C 1s and O 1s.^37^ This is further confirmed by the fact that the N 1s peak does not show any shift in binding energy, nor does it show any broadening (Figure S3B,C in the Supporting Information). Although the high binding energy of 288.4 eV of the smallest C 1s peak suggests a carboxylate (−CO_2_^–^) or a carbonate (−CO_3_^–^), the total amount of oxygen present in the O 1s peak speaks against this interpretation. The ratio of the total peak area of carbon, bound to oxygen, to the O 1s peak is closer to 1.4:1, which makes a ketone or aldehyde more likely to correspond to this high binding energy. The observed carbon bound to oxygen is higher than expected still, which could possibly result from the higher kinetic energy and thus larger probing depth of C 1s compared to the O 1s photoelectrons. The small (6% of total O 1 area) peak at a binding energy of 529 eV is a metal oxide, and the likely candidate is lead oxide (PbO), as lead dioxide (PbO_2_) would appear as a broader peak due to the presence of a strong satellite at higher binding energies.^38,39^ Because of the low amount of PbO, as suggested by the size of the peak in the O 1s spectrum, it cannot be observed in the Pb 4f spectrum, as it overlaps with the much stronger Pb–Br peak. The ratio of the relevant peak area in the O 1s spectrum and the Br 3d doublet peak area is ∼3:100 (Pb–O/Pb–Br).

**Table 1:** Measured Ratios of Spectrally Integrated XPS Peaks Divided by the Photoemission Cross Sections of the O 1s, C 1s, N 1s, Pb 4f, and Br 3d Core Levels, Normalized to the Spectrally Integrated Pb 4f Peak, for a Pristine and an XUV-Exposed Area of a MAPbBr_3_ Perovskite Sample

atom. species orbital	pristine	XUV exposed
Pb 4f	1	1
O 1s	0	1.1
C 1s	1.6	4.7
N 1s	0.8	0.8
Br 3d	2.4	2.3

**Figure 4:**
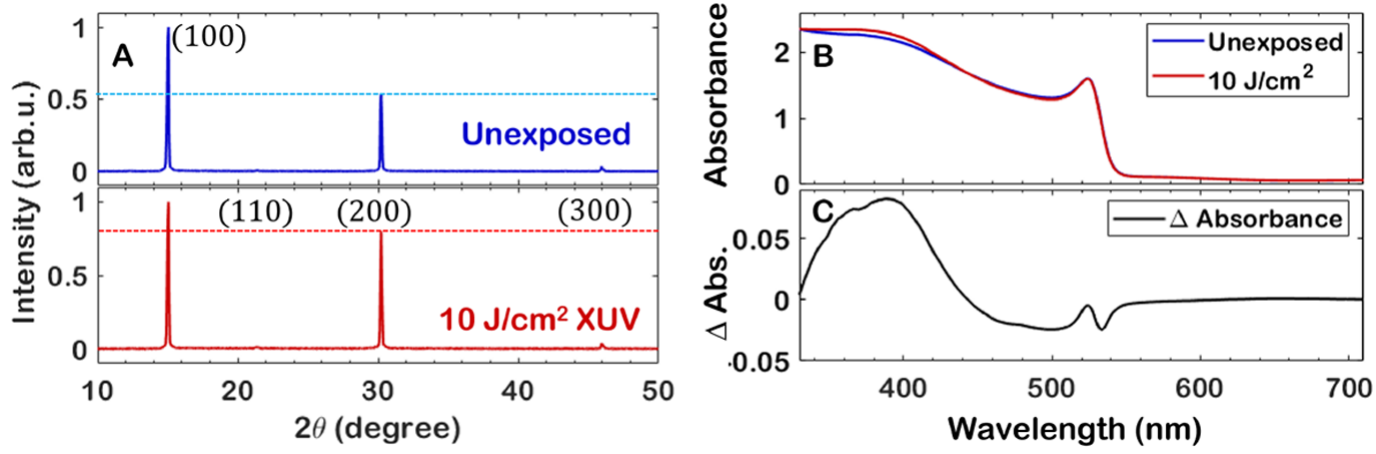
(A) XRD spectra of an unexposed and exposed area on the same sample of MAPbBr_3_ thin film, with peak positions in agreement with previously reported values^40^ but with vastly different ratios between the (100) and the (200) directions. (B) UV–Vis absorption spectra of an unexposed and exposed area. (C) Difference in absorbance between the exposed and unexposed areas, showing an increased absorbance around 395 nm.

XRD spectra show that the peak ratio between the (001) and (002) planes, which are normally approximately fixed over a chemically homogeneous sample, is significantly different when comparing an exposed and an unexposed part on the same sample, as can be seen in Figure 4A. Where the (002) peak is ∼50% of the height of the peak at (001) in the unexposed part of the sample, the exposed part shows a ratio closer to 80%, while for both the exposed and unexposed cases the 2θ angles agree with reported literature values.^40^ This again supports chemical change of the surface, rather than in the bulk, as the peaks do not broaden or shift from their 2θ angle. Indeed, formation or introduction of other atomic species at low percentages at the surface has been reported to change the ratio of XRD peaks without broadening or affecting the 2θ angle.^41,42^ A small change in UV–vis absorbance is also observable (Figure 4B,C), starting at ∼395 nm, as well as a small blue-shift of the exciton feature. This does not point to a particular species being formed but could result from the small amount of PbO that is formed, as the orthorhombic polymorph of PbO absorbs strongly in the blue to ultraviolet region and could thus also explain the small apparent blue-shift of the exciton feature.^43^

It is thus clear that the anomalous photostability in Figure 1C is caused by XUV induced passivation of the surface region, which removes trap states that would otherwise inhibit radiative decay of the excited state in untreated MAPbBr_3_. XUV radiation has a high enough photon energy to trigger the necessary chemical reactions. Nevertheless, at higher accumulated doses, the emission intensity still trends toward zero. For this reason, preoxidizing the surface can potentially also have a positive effect on the emission intensity over time, as trap states are removed before XUV excitation, instead of through XUV excitation. We explore this concept in the next section.

### Improved XUV Photostability through UV Ozone Treatment

**Figure 5:**
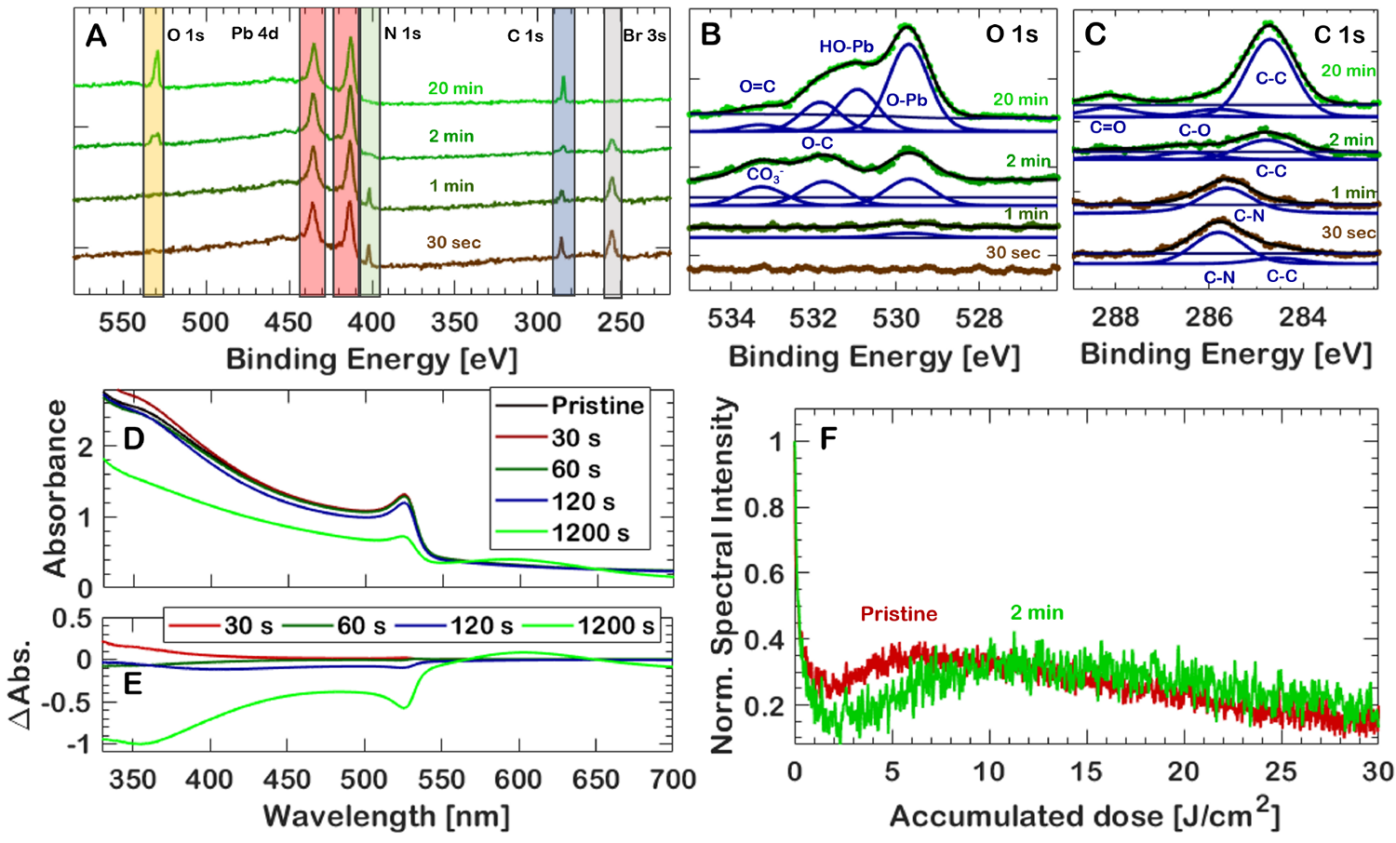
(A) XPS spectra of MAPbBr_3_ thin films at increasing UV ozone treatment durations. (B, C) O 1s and C 1s peaks of the ozone-treated samples with assigned species. (D) UV–Vis absorption of the ozone-exposed and unexposed samples. (E) ΔAbsorbance of ozone-treated samples. (F) Normalized PL intensities for a 120 s UV/ozone-treated and pristine thin film sample, in green and red, respectively, showing a significant shift in accumulated dose required to achieve the maximum intensity.

Samples of MAPbBr_3_ thin films were exposed to progressively longer durations of UV ozone treatment to passivate the surface before XUV excitation and to determine if there is an optimal ozone-treatment time before ozone starts negatively impacting XUV photostability through excessive oxidation. The results are summarized in Figure 5 and Table 2. The dose required to achieve maximum recovery increases when treating samples with ozone, at the cost of a slightly lower absolute PL intensity. This is apparent in Figure 5F: a near-doubling of the accumulated dose is required to achieve the maximum of the emission-intensity recovery, while it peaks to slightly lower values than before the sample was treated (from 35% to 32%). The minimum of the emission has decreased significantly (from 25% to 15%) after ozone treatment. The reason for this is unknown. The optimal time was found to be 120 s of ozone treatment—the associated curve is depicted in Figure 5F. Longer treatment times tend to have diminishing returns. See Figure S5B in the Supporting Information for the 10 min of UV ozone treatment case, showing both rapid photobleaching and no recovery as well as very little initial intensity to start with. No XUV-excited PL was observed in samples exposed to 20 min of UV ozone treatment, likely due to the ozone oxidizing the surface to such an extent and depth that the attenuation length of the XUV is comparable to the depth of oxidation, resulting in too few emitting states to be observed. This is also evidenced by the UV–vis absorption spectrum before and after ozone treatment, with a significant decrease in absorbance due to the oxidation in the case of 20 min of ozone treatment, as can be seen in Figure 5D. Despite the strong decrease in absorbance in the blue/green region of the spectrum, the increase in absorbance in the 580–630 nm region of the absorption spectrum actually causes samples with 20 min of UV ozone treatment to appear somewhat darker than before to the naked eye. The change is more clearly visible in the differential absorbance spectrum in Figure 5E. This absorption feature originates from the formation of PbO, which absorbs strongly around 600 nm in one of its polymorphs.^43^ Ozone treatment does not affect the electronic structure of the emitter under XUV excitation, as only the surface region of the material is affected. This can be seen in Figure S4A.

**Table 2:** Measured Ratios of Spectrally Integrated XPS Peaks at the O 1s, C 1s, N 1s, Pb 4f, and Br 3d Binding Energies, Normalized to the Pb 4f peak, for Increasing UV Ozone-Treatment Times^a^

atom. species	30 s	1 min	2 min	20 min
orbital				
Pb 4f	1	1	1	1
O 1s	0	0.05	0.5	1.2
C 1s	1.2	0.9	0.6	1.9
N 1s	0.8	0.7	>0^a^	0
Br 3d	2.5	2.3	1.2	0.1

a The ratio of N to Pb is larger than zero, as the peak is somewhat visible, but it cannot be properly fitted for quantification.

The exact chemical reactions occurring at the surface due to ozone treatment are different from those occurring due to XUV exposure, as evidenced by XPS measurements. This is not unexpected, as oxidation takes place due to an atmosphere of highly reactive ozone, instead of reactions with a thin water layer mediated by XUV photons. The XPS spectra of the ozone-treated samples in the range between binding energies of 580 and 220 eV are shown in Figure 5A, normalized to the spectrally integrated Pb 4f peak in the full spectrum (Figure S4A). Ratios of elements, normalized to Pb 4f, can be seen in Table 2. Looking at only the full spectrum and Table 2, several trends become apparent. First, the Pb 4f peak becomes broader as Pb–O bonds are formed and Pb–Br bonds are oxidized. The Pb 4f peak shows a small shift (Figure S4B,C), most apparent after 20 min of ozone treatment, due to the formation of PbO. The nitrogen bound in methylammonium is initially stable as evidenced by the presence of a C–N peak in the C 1s spectrum in Figure 5C and the single peak in the N 1s spectrum (Figure S4D), but after only 2 min of ozone treatment the remaining amount of nitrogen can no longer be properly quantified in either the C 1s or N 1s spectra, and after 20 min it is no longer measured. Bromine follows the same trend: it remains stable after up to 1 min of ozone treatment but disappears rapidly at longer treatment times, with approximately half remaining after 2 min and almost completely disappearing after 20 min (Figure S34E–G). Ozone treatment also causes an oxygen peak to appear after 2 min, as can be seen in detail in Figure 5B.

The C–C peak at low binding energies in the C 1s spectrum (Figure 5C) decreases faster than this O 1s peak appears, leading us to conclude that an ozone treatment first of all removes hydrocarbons from the surface before reacting with the surface itself. The O 1s peak becomes rather complex after only 2 min of UV ozone treatment, requiring at least three Voigt profiles to fit in the case of 2 min and four profiles in the case of 20 min of UV ozone treatment. The O 1s peak at 20 min of UV ozone treatment matches the O 1s peak reported by Wei et al. in case of their ozone oxidation treatment.^14^ The lowest-energy species at 529.5 eV is again PbO, which is much stronger than in the XPS spectrum of XUV-exposed MAPbBr_3_ in Figure 3B. It is unlikely to correspond to PbO_2_, as the ratio of oxygen present in the O 1s peak is closer to 0.8:1 compared to the total Pb 4f peak area. The slightly mismatched O–Pb/O ratio is possibly caused by the Pb 4f peak corresponding to two species, PbO and lead hydroxide (Pb(OH)_2_), which sometimes occurs as a shoulder-like peak in PbO XPS spectra.^38,44^ The ratio of the O 1s peak at a binding energy of 530.9 eV to the smaller Pb 4f peak (Figure S4B, top spectrum) is ∼2:1, which also supports this assignment, although it is unknown why there is no significant hydroxide peak in the case of 2 min of ozone treatment. The remaining two peaks in the O 1s spectrum are again oxidized carbon species, as there are matching peaks present in the C 1s spectrum. The lower binding energy peak matches to an equivalent peak in the carbon spectrum in an almost 1:1 ratio in both cases, suggesting the formation of a C–O bond, perhaps again in the form of an alcohol. A rather puzzling difference occurs in the case of the highest energy peak, however. The ratio of the C 1s peak at 288.4 eV to the O 1s peak at 533.3 eV is closer to 1:3 in the case of 2 min of UV ozone treatment, suggesting that a carbonate is formed. This changes in the case of a 20 min treatment, where the ratio is again closer to 1:1, suggesting a ketone or aldehyde to have formed concurrently with the lead hydroxide. The reason for this change is unknown but is perhaps linked to the disappearance of Br and the formation of lead hydroxide.

Overall, this leads us to conclude that an ozone treatment first oxidizes C–C species on the surface and thus cleans and perhaps already partially passivates the surface, which explains the reduction of the C 1s signal at 284.6 eV for short ozone-treatment times. After a tipping point between one and 2 min of ozone treatment, the ozone starts to react with nitrogen and bromide, causing outgassing, and the lead, alongside remaining carbon as well as additional carbon from the environment, is oxidized to form PbO and a series of oxidized carbon species. This simple demonstration thus shows that a UV ozone treatment is beneficial for indirect XUV scintillation in MAPbBr_3_ thin films but only up to a certain duration of UV ozone treatment that is yet to be properly determined and warrants further investigation.

## Conclusions

We have demonstrated that MAPbBr_3_ has remarkable XUV-excited emission properties in terms of emission intensity as a function of exposure time. Time-resolved XUV-excited luminescence measurements showed accelerated decays compared to UV-excited luminescence, which we attributed to an increased carrier concentration at the surface despite low excitation fluences. This increased carrier density drives photoinduced oxidation and photobleaching. The oxidation counteracts the photobleaching, and thus we observed a recovery of emission intensity as a function of exposure dose. XPS measurements helped attribute this recovery to the formation of passivating oxidized lead and carbon compounds. In order to harness this effect for improving the XUV-excited luminescence of MAPbBr_3_, we applied a simple ozone treatment to preoxidize the sample. This controlled passivation improved the photobleaching tolerance of our samples for XUV excitation, doubling the dose required to photobleach the sample completely. Our results demonstrate that MAPbBr_3_ can be used as an indirect XUV scintillator, due to its remarkable recovery mechanism, especially when pretreated with ozone.

## Supporting Information Available

Additional XPS spectra, carrier density calculation details as well as estimates of XUV excited PL intensity as a function of irradiation dose and XUV excited PL intensity during intermittent exposure (PDF)

## Notes

**Data****availability**. The data that support the findings of this study are available from the corresponding author upon reasonable request.
