# Supplementary material for: Extreme-Ultraviolet Excited Scintillation of Methylammonium Lead Bromide Perovskites
Source: J Phys Chem C Nanomater Interfaces. 2022 Jul 21;126(30):12554–62. doi: 10.1021/acs.jpcc.2c02400 (PMC9358647; doi:10.1021/acs.jpcc.2c02400)
Supplement: Supplementary file 1 — jp2c02400_si_001.pdf [file jp2c02400_si_001.pdf]

Supporting Information for Publication: Extreme-Ultraviolet Excited Scintillation of Methylammonium Lead Bromide Perovskites

Maarten L.S. van der Geest,[†] Lucie McGovern,[‡] Stefan van Vliet,[†] Hanya Y. Zwaan,[†] Gianluca Grimaldi,[‡,¶] Jeroen de Boer,[‡] Roland Bliem,[†,§] Bruno Ehrler,[‡] and Peter M. Kraus[*,†,‖]

†*Advanced Research Center for Nanolithography (ARCNL), Science Park 106, 1098 XG, Amsterdam, The Netherlands.*

‡*Center for Nanophotonics, AMOLF, Science Park 102, 1098 XG, Amsterdam, The Netherlands.*

¶*Cavendish Laboratory, University of Cambridge, Cambridge CB2 1TN, United Kingdom*

§*Institute of Physics, University of Amsterdam, Science Park 904, 1098 XH Amsterdam, The Netherlands*

‖*Department of Physics and Astronomy, and LaserLaB, Vrije Universiteit, De Boelelaan 1105, 1081 HV Amsterdam, The Netherlands.*

E-mail: p.kraus@arcnl.nl

Intermittent Exposure

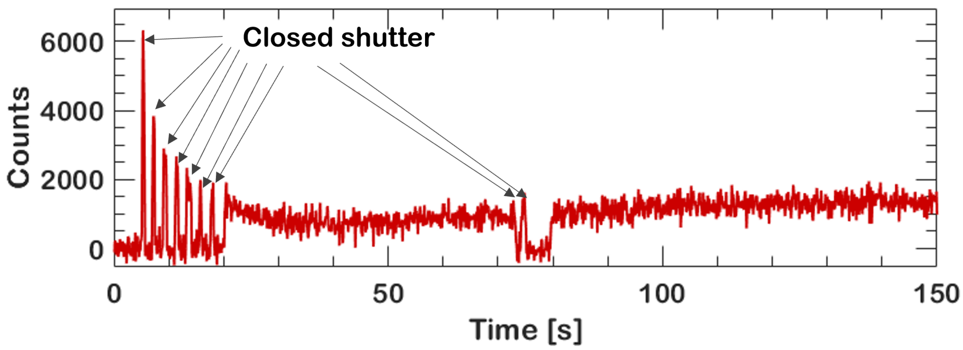

Figure S1: Number of counts measured as a function of time in case of XUV exposure of methylammonium lead bromide with intermittent opening and closing of the shutter to block the XUV light. The graph indicates that no intermediate recovery occurs when the shutter is closed. The PL intensity continues as if the shutter never closed.

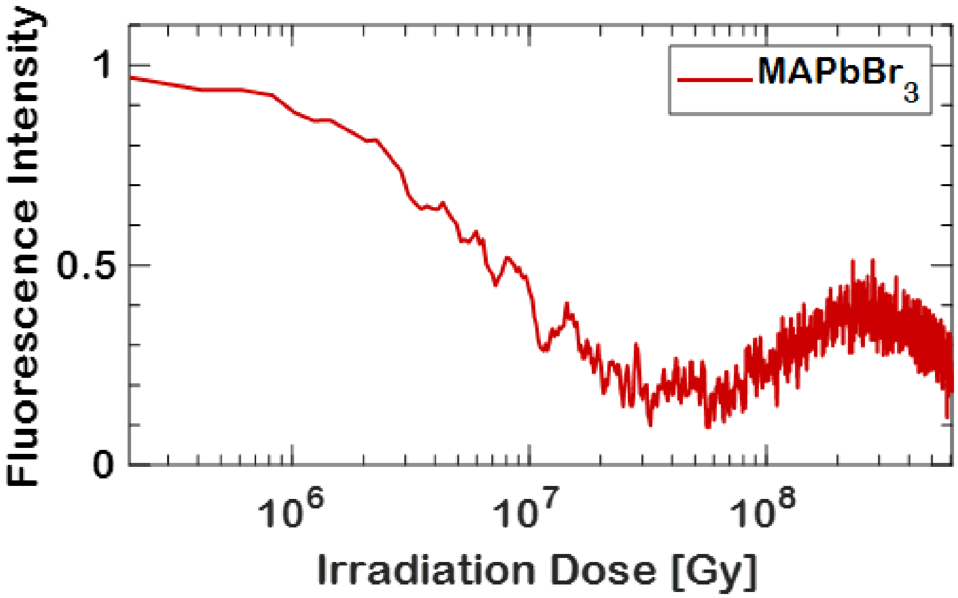

Figure S2: Using an attenuation length of 75 nm (99%) absorption and a density of 3.5 g/cm^3,[1] the dose axis can be converted to an approximate absorbed irradiation dose in Gray y (J/kg)to allow a tentative comparison with other reported scintillator photobleaching curves. It is non-trivial to compare the photobleaching rate with published bleaching rates of common scintillators, such as anthracene or plastic scintillators, due to sharp contrasts in attenuation length across the XUV spectrum used as bleaching source in our experiments, as can be seen in Fig. 1A of the main text, when compared to hard X-rays. Nevertheless, when the dose axis is converted the photobleaching performance is comparable to several plastic scintillators.[2]

Additional XPS data to section 3.3

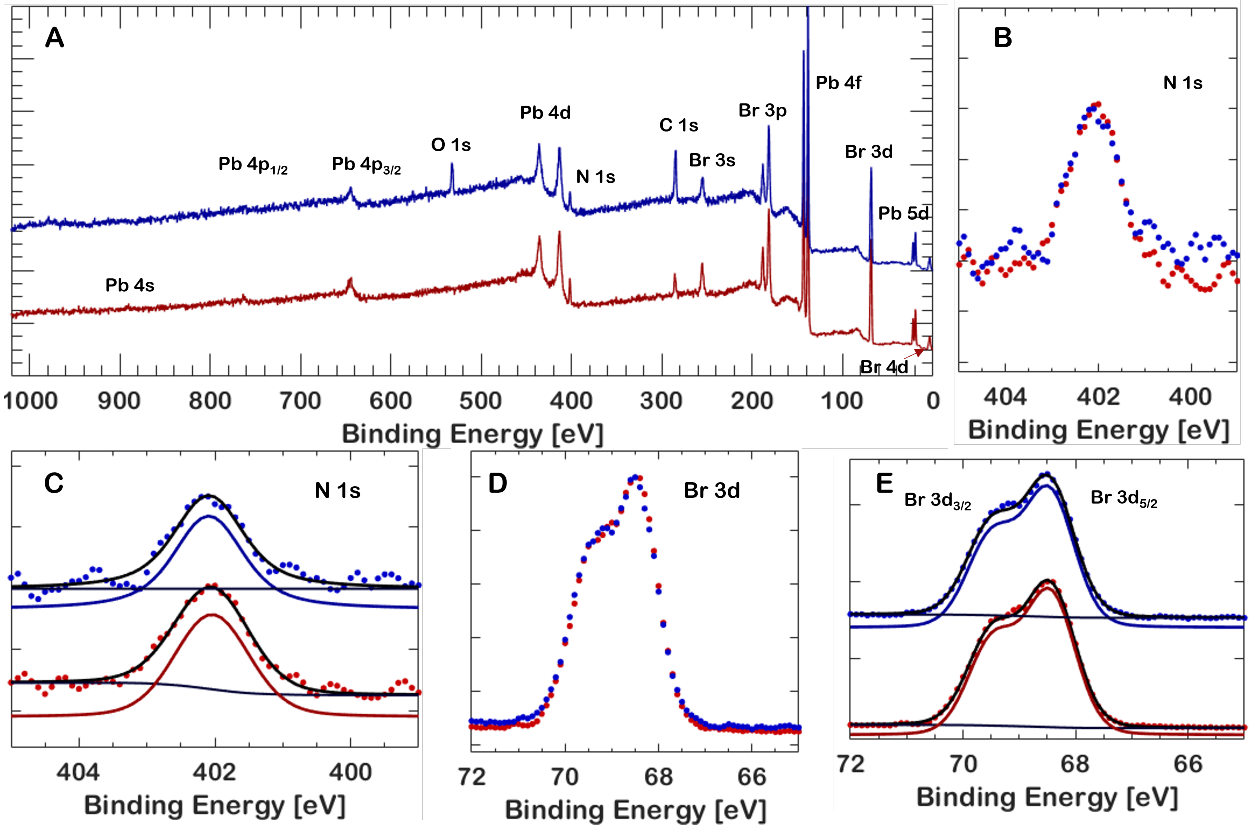

Figure S3: A) Full survey of Fig.3A in the main text with all visible peaks labeled B) N 1s peaks in exposed and unexposed area of sample overlaid C) N 1s Fitted D) Br 3d in exposed and unexposed area of sample overlaid E) Br 3d peak fitted

Additional XPS data to section 3.4

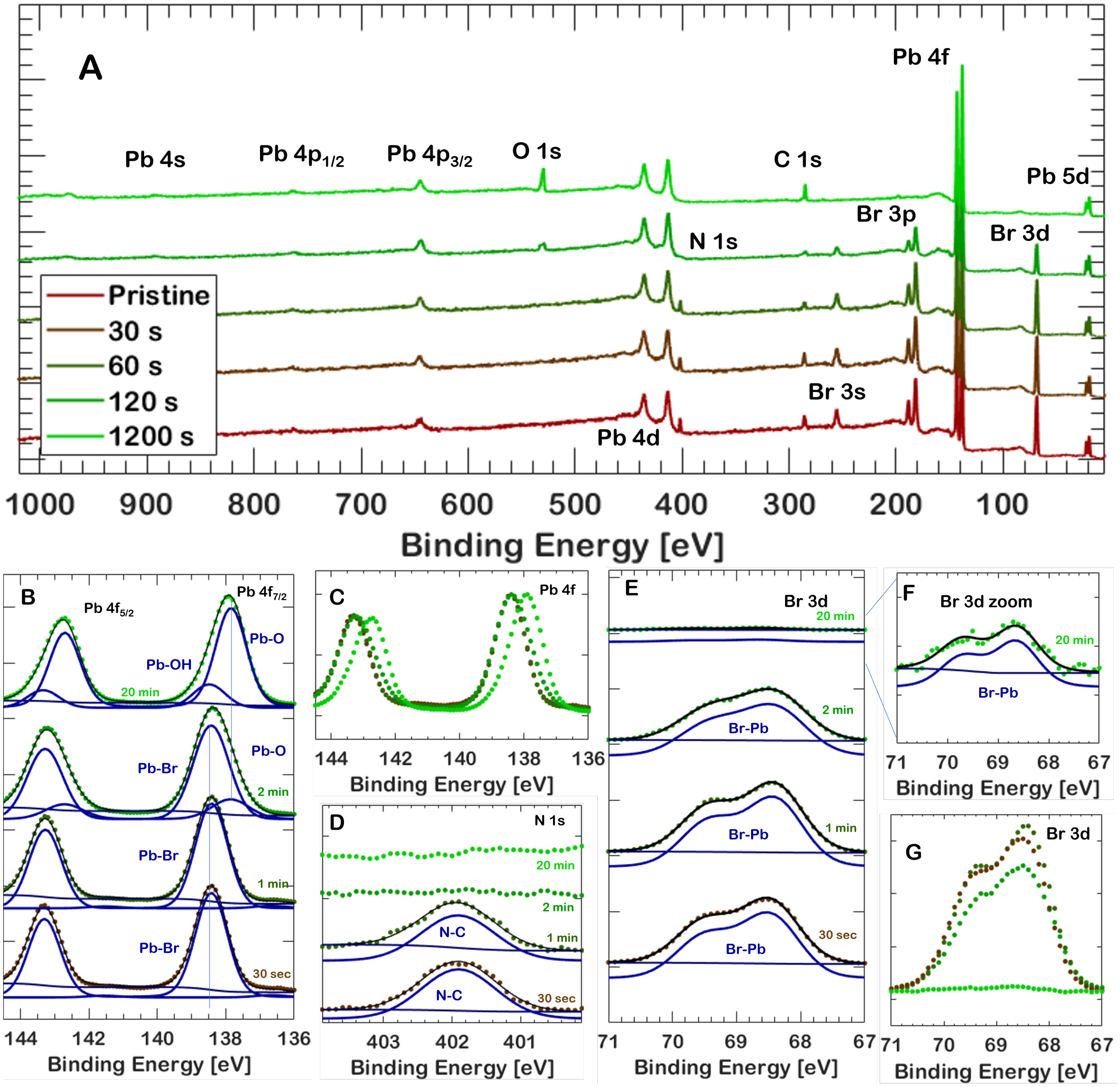

Figure S4: A) Full survey of Fig.5A in the main text with all visible peaks labeled B) Pb 4f peaks fitted C) Pb 4f overlaid D) N 1s peaks fitted E) Br 3d peak fitted F) Br 3d zoom for 1200 s G) Br 3d overlaid

Spectral data Ozone treatment

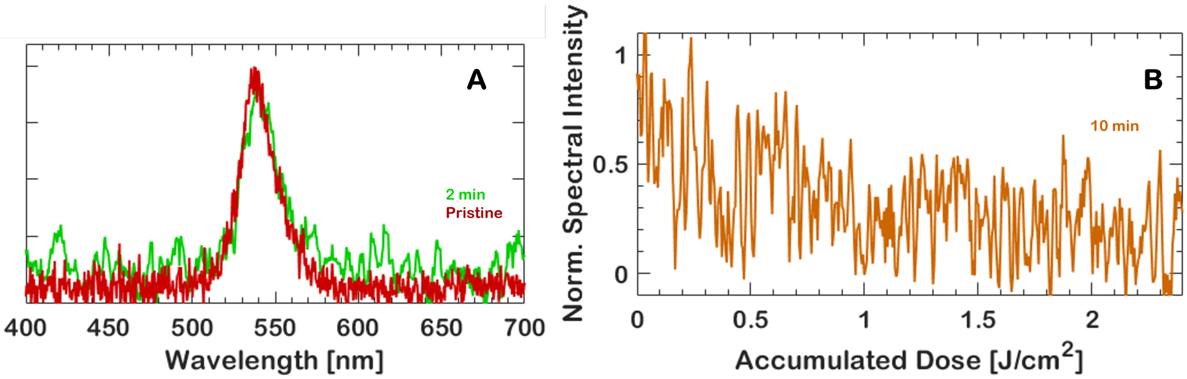

Figure S5: A. Emission profile accompanying normalized spectral intensities in Fig.5F B. Normalized spectral intensity excited with broadband, pulsed XUV radiation, measured after ten minutes of ozone exposure

Estimating carrier densities in $MAPbBr_3$

Pulsed DUV & visible excitation

The carrier densities n (cm^{-3}) in $MAPbBr_3$ were estimated based on the measured absorption spectrum in Fig.1B and an estimate of the reflection based on tabulated complex refractive index values. A similar method has been described by Cushing *et al.* to estimate carrier densities in silicon (100).[3] The absorbance A_λ for the excitation wavelengths used in the manuscript were $A_{260} = 2.2$, $A_{330} = 2.2$ and $A_{400} = 2.1$, respectively. Thus, more than 99% of all incoming photons are absorbed or reflected in all cases and we assume that all light is either absorbed or reflected. As the exact thickness of the area probed with ultraviolet-visible absorption spectroscopy was not known, we use the tabulated absorbance for further improved accuracy.[4] The refractive indices of $MAPbBr_3$ n_λ are $n_{260} = 2.3$, $n_{330} = 2.3$ and $n_{400} = 2.3$, respectively, while the complex refractive indices k_λ are $k_{260} = 0.16$, $k_{330} = 0.67$ and $k_{400} = 0.39$, respectively as measured by Park *et al.*[4] Vacuum has, by definition, $n = 1$ and $k = 0$ for all wavelengths. As the excitation occurs under normal incidence, the power reflection coefficient $\mathbf{R}$ on the boundary between vacuum (medium 1) and perovskite (medium

2) can thus be found using the simplified Fresnel equation, Equ.:

$$\mathbf{R} = \left|\frac{n_1 - n_2}{n_1 + n_2}\right|^2 = \frac{(1 - n_{\mathrm{MAPbBr_3}})^2 + k^2}{(1 + n_{\mathrm{MAPbBr_3}})^2 + k^2} \tag{S5}$$

This yields power reflectances of $\mathbf{R}_{260}$= 0.16, $\mathbf{R}_{330}$= 0.19 and $\mathbf{R}_{400}$= 0.17, respectively at the vacuum/perovskite boundary. We are furthermore assuming a homogeneous, Gaussian beam profile and neglecting further losses due to scattering. Due to our neglecting of scattering, the actual carrier density will be lower. Amplified spontaneous emission is clearly visible in the case of 330 nm and 400 nm excitation, so a carrier concentration above 10^{19} cm^{-3} is expected.[5] The excitation pulses, originating from the optical parametric amplifier, have durations between 60 and 80 femtoseconds as measured with frequency resolved optical gating. We thus assume that the carrier distribution appears in an instant in the material and returns to equilibrium through radiative and non-radiative processes on a much longer time scale.

The recorded single pulse fluence in the case of 330 nm excitation was 17 $\mathrm{mJ/cm^2}$ which is contained within the 150 μm full-width at half maximum (FWHM) of the Gaussian beam profile (which contains 76% of total fluence). Furthermore, it is assumed that 330 nm photons cannot excite more than one carrier per photon and that leftover energy is lost through non-radiative decay. The pulse contains $2.8 * 10^{16}$ $\mathrm{phot/cm^2}$, calculated by converting the photon energy and fluence to electronvolts. Taking into account that 19% of these photons are reflected and not taking into account any additional scattering losses, we take the average sample thickness to find a maximum carrier concentration of $9.8 \pm 0.1 * 10^{20}$ cm^{-3} across the sample thickness.

For an excitation fluence of 8 $\mathrm{mJ/cm^2}$/pulse in the case of 400 nm. Using the same method as before, one finds $1.7 * 10^{16}$ $\mathrm{phot/cm^2}$ and carrier density of approximately $6.7 \pm 0.2 * 10^{20}$ cm^{-3}. In the case of 260 nm excitation, the excitation fluence was 50 $\mu\mathrm{J/cm^2}$/pulse. This yields a much lower $6.5 * 10^{13}$ $\mathrm{phot/cm^2}$ and consequently a lower estimated carrier concentration of $3.0 \pm 0.3 * 10^{18}$ cm^{-3}.

Pulsed XUV excitation

In the case of XUV excitation, one can use the same method, but one has to take into account the broadband energy range ($20 - 45$ eV) of the XUV source and the large change in absorbance across that same range (blue curve in Fig.1a). The estimated single pulse fluence across the XUV range is approximately 26 μJ/cm^2 and the distribution of photons across photon energies $\rho(eV)$ corresponds to the red curve in Fig.1A. The XUV absorbance of $MAPbBr_3$ was calculated using atomic scattering factors[6] and as such can be converted to an energy dependent [nm^{-1}] absorbance ($\alpha(eV)$).

One then takes the previously calculated photon distribution and propagates it through the thin film with the theoretical absorbances according to Equ.S5 to get a carrier distribution $n(eV)$:

$$n(eV) = \rho(eV) * 10^{-\alpha(eV)} \tag{S5}$$

This yields the distribution in Fig.S6, depicting two possible extremes. One in which one photon creates one carrier (blue curve) and one in which the photon energy is converted in the maximum possible amount of discrete carriers, rounded down (red curve). This unknown conversion factor of photons to carriers is the reason for the large range between which the carrier density can lie.

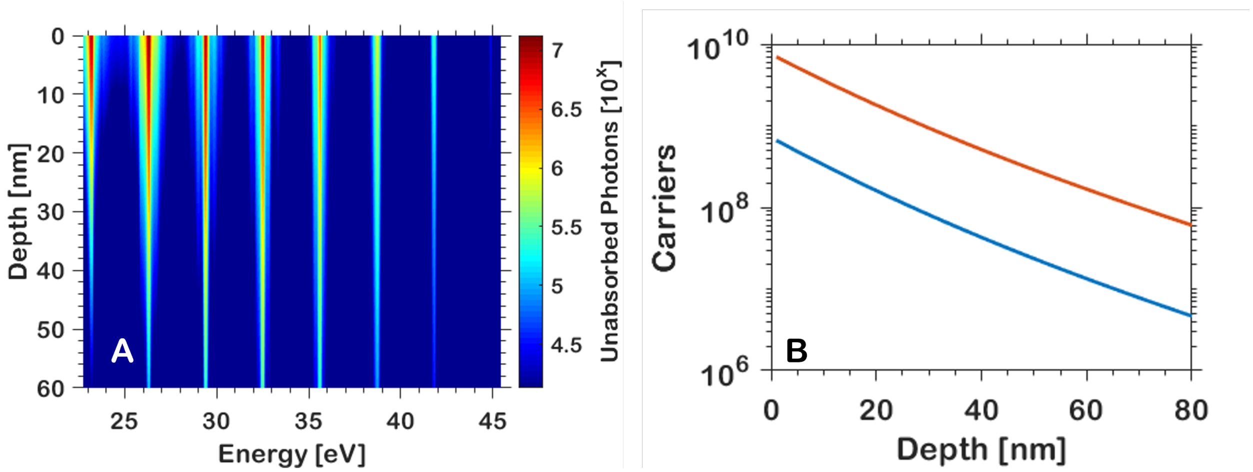

Figure S6: A) Propagating XUV light through nm's of $MAPbBr_3$ as a function of photon energy. Colorbar indicates powers of ten. B) Number of carriers as a function of depth over the entire spotsize, varying between a low estimate of one carrier/photon (blue curve) to the maximum possible where all photon energy is converted inelastically into carriers (red curve) with an energy of 2.53 eV (exciton energy). The sum of carriers between two depth boundaries can be converted to a carrier density estimate by dividing by the spotsize.

This model thus predicts a carrier density in the surface region (first 10 nm) of $2.3 * 10^{20}$ to $2.4 * 10^{21}$ cm^{-3}. The average carrier density up to the 99% absorbance depth (first 75 nm) similarly ranges from $6.2 * 10^{19}$ to $6.7 * 10^{20}$ cm^{-3}.

References

(1) Birowosuto, M. D.; Cortecchia, D.; Drozdowski, W.; Brylew, K.; Lachmanski, W.; Bruno, A.; Soci, C. X-ray scintillation in lead halide perovskite crystals. *Sci. Rep.* **2016**, *6*, 1–10.

(2) Jivan, H.; Mdhluli, J. E.; Sideras-Haddad, E.; Mellado, B.; Erasmus, R.; Madhuku, M. Radiation damage effects on the optical properties of plastic scintillators. *Nucl. Instrum. Meth. B* **2017**, *409*, 224–228.

(3) Cushing, S. K.; Porter, I. J.; de Roulet, B. R.; Lee, A.; Marsh, B. M.; Szoke, S.;

Vaida, M. E.; Leone, S. R. Layer-resolved ultrafast extreme ultraviolet measurement of hole transport in a Ni-TiO2-Si photoanode. *Sci. Adv.* **2020**, *6*, eaay6650.

(4) Park, J. S.; Choi, S.; Yan, Y.; Yang, Y.; Luther, J. M.; Wei, S. H.; Parilla, P.; Zhu, K. Electronic structure and optical properties of α-CH3NH3PbBr3 perovskite single crystal. *J. Phys. Chem. Lett.* **2015**, *6*, 4304–4308.

(5) Richter, J. M.; Abdi-Jalebi, M.; Sadhanala, A.; Tabachnyk, M.; Rivett, J. P.; Pazos-Outón, L. M.; Gödel, K. C.; Price, M.; Deschler, F.; Friend, R. H. Enhancing photoluminescence yields in lead halide perovskites by photon recycling and light out-coupling. *Nat. Commun.* **2016**, *7*, 1–8.

(6) Henke, B. L.; Gullikson, E. M.; Davis, J. C. X-ray interactions: Photoabsorption, scattering, transmission, and reflection at E = 50-30, 000 eV, Z = 1-92. *Atom. Data Nucl. Data* **1993**, *54*, 181–342.
